# Quality Assessment of YouTube Videos on Complementary and Alternative Medicine (CAM) for Cancer Using a Newly Developed Tool

**DOI:** 10.1177/15347354241293417

**Published:** 2024-10-28

**Authors:** Sophia Huchel, Alina Grumt, Christian Keinki, Judith Buentzel, Lukas Käsmann, Jutta Huebner

**Affiliations:** 1 University Hospital Jena, Jena, Germany; 2 University Medical Center Goettingen, Goettingen, Germany; 3 University Hospital, LMU Munich, Munich, Germany; 4 German Center for Lung Research (DZL), Partner Site Munich, Munich, Germany; 5 German Cancer Consortium (DKTK), Partner Site Munich, Munich, Germany

**Keywords:** complementary and alternative medicine (CAM), cancer, YouTube, assessment tool, health literacy, videos, quality

## Abstract

**Background:**

The global burden of cancer continues to rise and complementary and alternative medicine (CAM) is attracting a lot of interest. However, quality of online information on CAM, particularly on platforms like YouTube, remains questionable. This study aimed to create a comprehensive assessment tool to assess the quality of CAM-related YouTube videos, crucial for informed decision-making in oncology.

**Methods:**

The assessment tool was developed by adapting existing criteria for website content analysis to video rating. A YouTube search was conducted using German-language terms related to CAM (“complementary medicine (CM) for cancer” and “alternative medicine (AM) for cancer”). In total 25 videos were assessed based on the defined criteria and assigned to five different types of providers (journalism, healthcare organization, hospital or health insurance, independent person, non-medical organization). Statistical analysis was conducted using IBM SPSS Statistics 27.

**Results:**

Interrater reliability analysis showed an Intraclass Correlation Coefficient (ICC) of .91, indicating good to excellent agreement. The average video result was of poor quality, with none of the videos meeting all criteria. The videos achieved a mean rating of 38.2 points (SD: 6.5 points; possible range: 20-60 points). Journalism-based videos showed the most views per days, particularly surpassing those from hospitals or health insurance providers (Kruskal-Wallis-Test: *z* = 3.14, *P* = .02). However, there was no statistically significant correlation between video quality and the type of provider or interaction indices. Videos retrieved under the search term “CM” generally scored higher in quality than those under “AM” (Mann-Whitney U test: *U* = 39.5, *P* = .04). Nonetheless, "CM" videos were less frequently viewed (Mann-Whitney U test: *U* = 31.0, *P* = .01).

**Conclusion:**

This study, the first of its kind focusing on CAM in cancer care emphasized the challenges in identifying credible sources on social media platforms such as YouTube. The developed assessment tool offers a more detailed evaluation method for health-related videos but requires further refinement and testing. Collaboration between healthcare and media entities is suggested to improve the dissemination of reliable information on platforms like YouTube.

## Introduction

Worldwide, nearly 20 million people developed cancer in 2022, and nearly 10 million cancer-related deaths were attributed.^1^ The number of cases is rising steadily, with the World Health Organization (WHO) predicting more than 32 million new cases by 2045.^2^ In Germany, as well as worldwide, cancer is the second leading cause of death, after diseases of the circulatory system.^3,4^ With cancer accounting for such a large proportion of all diseases and disease-related deaths, alternative treatments are gaining more and more interest. A cancer diagnosis often leaves patients feeling powerless and, consequently, with a need to take some kind of action against the disease themselves.^5^ This is the time when they may turn to complementary and alternative medicine (CAM) because here they may act for themselves and increase a sense of self-efficacy. CAM comprises divided allcomplementary medicine (CM) and alternative medicine (AM). However, based on the definition used, CM and AM vary significantly. Using a common definition, CM stands for procedures that complement conventional medicine, such as Qigong for cancer patients with insomnia.^6^ AM stands for methods or medication aiming to achieve cure by methods lacking evidence of benefits being greater than harms. In addition, AM may result in postponing or declining evidence-based conventional treatment.^7^

According to various patient surveys, the most common sources of information for cancer patients are talking directly to their doctor, followed by support groups and print media, and the Internet as the third most common source of information.^8,9^ In contrast, studies show that while 1 in 2 cancer patients uses CAM methods,^10-15^ around 40% of patients do not tell their healthcare professionals.^12,16-19^ Patients perceive doctors as not competent enough when it comes to CAM^12,20^ and are confronted with a lack of time during the doctor-patient consultation.^12^ YouTube is the second most accessed website and therefore the most used video platform worldwide.^21^ The same applies to Germany as a single country.^21^ In Europe, a report published in 2014 found that about 60% of the population searched for health information on the Internet within a year.^22^ And since then, Internet use has increased rapidly.^23^ YouTube is an easy-to-use video platform that allows both uncomplicated uploading and consumption of video material all over the world. Of course, this poses difficulties, such as the lack of verification of uploaded information for example, through quality seals, which can easily lead to the spread of misinformation such as the fenbendazole scandal in 2020.^24^ In the case of CAM and oncology this can even lead to patients postponing curative treatments, ingesting toxic substances or risking uncontrolled drug interactions.^7,12,24,25^ Especially when it comes to health information, users quickly feel insecure and overwhelmed by the endless abundance of videos and do not know how to identify the videos that are suitable and trustworthy.^26,27^

For this very reason, our aim was to take a closer look at the quality of information regarding CAM on YouTube and find ways to identify high-quality videos using a standardized assessment tool. As present tools for assessing video quality mostly focus on a few specific criteria, our aim was to create a comprehensive tool that includes all main aspects contributing to quality and reliability.

## Methods

### Development of the Assessment Tool

#### Analysis of existing assessment tools for written information

Our starting point was a detailed analysis of an already existing assessment tool for medical content on websites, designed by our working group.^28^ In the meantime, there is also a review paper by Josfeld and Huebner on the analysis of assessment tools for different types of patient information.^29^ Liebl et al^28^ described the compilation of rating tools for website content based on national as well as international criteria and recommendations for patient information: Health On The Net code of conduct (HONcode),^30^ DISCERN,^31^ guidelines of the Agency for Quality in Medicine (ÄZQ),^32^ criteria for transparency of the Action Forum on Health Information Systems (afgis)^33^ and publications of Steckelberg et al^34^ HONcode represents a selection of criteria for providers of health information on the Internet and issues the HONcode certificate. DISCERN is a tool that helps patients determine what information they can trust.^35^ The guidelines of the ÄZQ and literature by Steckelberg et al are evidence-based recommendations on patient information. The afgis criteria for transparency are provided by the German Action Forum on Health Information Systems and identify websites that offer high-quality health information with the focus on transparency.

#### Adaptation of the criteria to the assessment of visual media

Three of the authors (SH, AG, JH) revised the above tools, removed criteria that were not transferable to the assessment of videos or aligned criteria with video rating to shift the focus from written information to auditory and visual information. Since essential criteria, such as audio quality, were not yet represented, we analyzed further assessment tools to improve our tool and adjust it to video rating. Usefulness score,^36^ accuracy score,^37^ Video information and quality index (VIQI) Scale^38^ and Medical information and context index (MICI)^38^ either duplicated existing criteria, were very specific in content, or were not transferable to videos, so they could not show us any new criteria. The Title-content-consistency index (TCCI)^39^ rates the sensationalist style of a video by pointing out how the title of the video matches the content. The original is a 5-point Likert scale. To adapt it to our tool we have modified and added it to the criterion “video description.” Patient Education Materials Assessment Tool (PEMAT)^40^ functions as a guide to analyze whether patients are capable of understanding and responding to information. From this, we have taken the criteria of comprehensibility and actionability and aids for evaluating the audio and visual quality of the videos. In addition, we took the criterion “authorship” from an article by the Journal of the American Medical Association (JAMA)^41^ to complement the criterion “transparency.” In total, the assessment tool is composed of 3 main criteria: Content, user-orientated and formal criteria, and its twenty sub-criteria. An overview of the criteria and their origin is provided in Table 1. Finally, we conducted 3 test runs to review and revise the instrument with the aid of 3 physicians, a research assistant, a patient representative, 2 medical students and 4 laypeople. The final assessment tool is provided in Appendix Table A1.

**Table 1.** Criteria for the Assessment of Videos and Their Origin.

Content criteria		User-orientated criteria		Formal criteria	
Goal	Liebl et al. (DISCERN)	**Relevance**	Liebl et al. (DISCERN, ÄZQ)	**Transparency**	Liebl et al. (DISCERN, HONcode, afgis), JAMA
Clarity	Liebl et al. (Steckelberg et al.), PEMAT	**Feasibility**	PEMAT	**References**	Liebl et al. (DISCERN, HONcode, afgis)
Comprehensibility of structure	Liebl et al. (ÄZQ, Steckelberg et al.), PEMAT	**Interaction with users**	Liebl et al. (afgis)	**Quality assurance procedure**	Liebl et al. (afgis), JAMA
Comprehensibility of language	Liebl et al. (Steckelberg et al.), PEMAT	**Video description**	TCCI	**Pointing out limitations of the medium**	Liebl et al. (HONcode)
Detailed information about treatment methods	Liebl et al. (DISCERN, Steckelberg et al.)			**Audio quality**	PEMAT
Completeness	Liebl et al. (DISCERN, ÄZQ)			**Visual quality**	PEMAT
Accuracy	Liebl et al. (ÄZQ, Steckelberg et al.)				
Presentation of numbers and outcomes	Liebl et al. (Steckelberg et al.)				
Evidence-based information	Liebl et al. (HONcode, Steckelberg et al.)				
Communication of missing evidence	Liebl et al. (DISCERN, Steckelberg et al.)				

Liebl et al,^28^ DISCERN,^31^ Steckelberg et al,^34^ PEMAT,^40^ ÄZQ,^32^ HONcode,^30^ afgis,^33^ JAMA.^41^

### Selection of YouTube Videos on CAM

**Figure 1.**
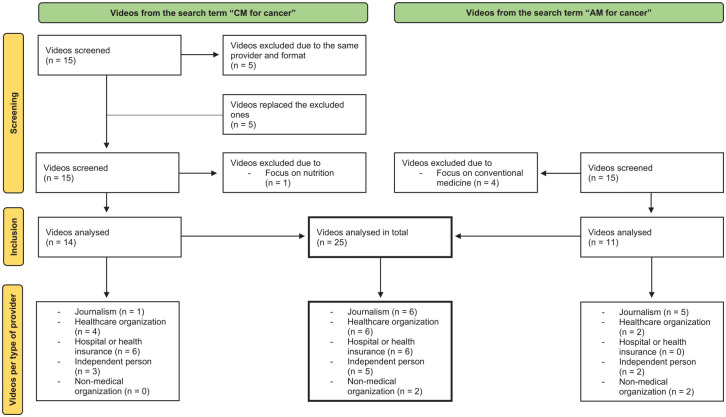
Overview of the selection of videos and classification into types of providers.

The YouTube search was conducted on February 24, 2022, using the German website and its basic settings (including e.g. relevance-based ranking). In order not to influence the search, we performed it on a public computer without pre-sets and cookies. We searched for “Komplementärmedizin bei Krebs” (in English „complementary medicine for cancer“) and “Alternativmedizin bei Krebs” (in English „alternative medicine for cancer“). The search was conducted in German, only reviewing German language videos that contained the searched topic in their titles. The further selection of the videos and the assignment to the corresponding type of provider was verified by AG and JH. Parallel to the national German guideline program, which plans to provide a guideline for CAM in cancer care and separate guideline for nutrition in cancer, we have excluded videos that mainly relate to nutrition. Further exclusion criteria were the duplication of videos from the same series with the same speaker and videos focusing on conventional medicine. Since research from Gudivada et al^42^ suggests that YouTube users tend to only look on the first page of results, we decided to include fifteen videos of each search term in our video assessment. Four videos from the search term “AM for cancer” were excluded due to their focus on conventional medicine. One Video from the search term “CM for cancer” was excluded due to its focus on nutrition. In 2 cases with the search term "CM for cancer", several videos of the same series and the same speaker would have been included in the rating. In these cases, we only considered the video that was displayed first and replaced the missing number of videos with the videos from the list of those that have moved up. After the exclusion of all inappropriate videos, we assessed 25 videos in total. We divided all videos into 5 types of providers, identifiable for laymen: Healthcare organization, hospital or health insurance, journalism, independent person and non-medical organization. “Healthcare organization” included self-help groups, health-related information centers, associations or academies. “Independent person” included all producers who stood for themselves, with or without medical qualification. “Non-medical organization” included all organizations and institutions without a specific medical background, for example, a publishing house. For more detailed information see Figure 1.

### Assessment of the Videos

All video links and video characteristics, such as the source, type, upload date, duration, number of views, number of comments, and number of likes were recorded for further analysis. We calculated the view ratio by dividing the number of views by the number of days the video has been online.^43^ The count of dislike was deactivated during our research.

Four of the authors, 2 physicians (JH, CK) and 2 medical students (SH, AG), assessed the videos independently and an arithmetic mean was generated to compare the videos with each other. The rating was done using a 3-point Likert scale: 3 = the video fulfills the criterion at its best, 2 = the video misses the criterion in some points, 1 = the video barely meets the criterion. The overall result to be achieved as the lowest was 20 points (=0%), the one to be achieved as the highest was 60 points (=100%). We classified the videos in 4 levels from very poor (0%-25%), poor (25%-50%), good (50%-75%) to very good (75%-100%).

### Statistical Analysis

IBM SPSS Statistics 27 was used for descriptive and inferential analysis. ICC (Intraclass Correlation Coefficient), mean, minimum, maximum, standard deviation and variance were calculated. As Shapiro Wilk normality test resulted in non-normally distributed data, the correlation between 2 independent numerical variables was calculated using Spearman’s nonparametric correlation coefficient (Spearman’s ρ). The relationship between groups and individual variables was calculated using the Kruskal Wallis test. To be able to make statements about the data for the 2 different search terms, the Mann-Whitney U test was performed. To determine interrater reliability, ICC and its 95% confidence interval were calculated based on mean-rating (*k* = 4), 2-way mixed-effects model and absolute agreement. It was interpreted with the help of Koo and Li’s recommendations.^44^ The statistical significance level was set at *P* < .05.

## Results

### Interrater Reliability of the Assessment Tool

An ICC with 0.91 (95 % confidence interval = 0.80-0.96) in the interrater reliability analysis indicates a good to excellent agreement according to Koo/Li.^44^ Looking at the ICC result in relation to the 2 different searches, “CM” had an ICC of 0.88 and “AM” of 0.91, both indicating a moderate to excellent agreement according to Koo/Li. An overview of the results, also in relation to the 2 rater groups, is provided in Table 2.

**Table 2.** Intraclass Correlation Coefficient (ICC) Looking at Either All Videos or Only Videos With the Search Term “CM” or Only Videos With the Search Term “AM” Rated by Either All Raters or Only Physicians or Only Students.

	All videos (n = 25)	Only videos with the search term “CM” (n = 14)	Only videos with the search term “AM” (n = 11)
All raters	0.91 [0.80, 0.96]	0.88 [0.72, 0.96]	0.91 [0.68, 0.98]
Only physicians (n = 2)	0.93 [0.83, 0.97]	0.87 [0.58, 0.96]	0.95 [0.11, 0.99]
Only students (n = 2)	0.90 [0.78, 0.96]	0.88 [0.64, 0.96]	0.90 [0.59, 0.97]

The confidence interval is shown in brackets.

### Videos and Their Characteristics

After we sorted out the inappropriate videos, we had fourteen videos to rate from the search term “CM” and eleven videos from the search term “AM.” We assigned six videos each to the group “journalism,” “healthcare organization” and “hospital or health insurance”. Five videos were assigned to the group "independent person" and two videos to the group "non-medical organization” (Figure 1). An overview of the video characteristics is provided in Table 3.

**Table 3.** Characteristics of the Videos (N = 25).

Video characteristics	Minimum	Maximum	Span	Mean	Standard deviation	Variance
Running time [seconds]	121.0	5231.0	5110.0	1859.6	1636.9	2679477.9
Views	57.0	401664.0	401607.0	51945.5	112959.1	12759761321.0
View ratio [views/day]	0.1	17049.2	17049.1	738.0	3400.5	11563222.5
Comments	0	2110.0	2110.0	171.8	464.1	215389.9
Likes	0	9996.0	9996.0	889.7	2228.4	4965598.2

### View Ratio and User Behaviour

A closer look at the view ratio (number of views per days the video is online) revealed a maximum of 17049.2 views/day, followed by 459.7 and 415.7 views/day. The videos with the 4 highest values in the view ratio (235.0-17049.2 views/day) were all from the group “journalism,” followed by the video in fifth position (106.8 views/day) from the group “healthcare organization.” The 5 videos with the lowest values (0.1-0.4 views/day) were 2 from the group “independent person” and 3 from the group “ hospital or health insurance.” The view ratio partially correlated significantly with the type of provider: videos from the group “journalism” were watched more often than videos from the group “hospital or health insurance” (Kruskal-Wallis-Test: *z* = 3.14, *P* = .02). This also applied if the most viewed video, which is an outlier, was removed from the test. Among the 5 highest values for view ratio, all videos came from the search term “AM” and all 5 videos with the lowest values for view ratio came from the search term “CM.” The Mann-Whitney U test showed that videos from the search term “AM” were watched significantly more often than videos from the search term “CM” (*U* = 31.0, *P* = .01).

### Overall Results

**Figure 2.**
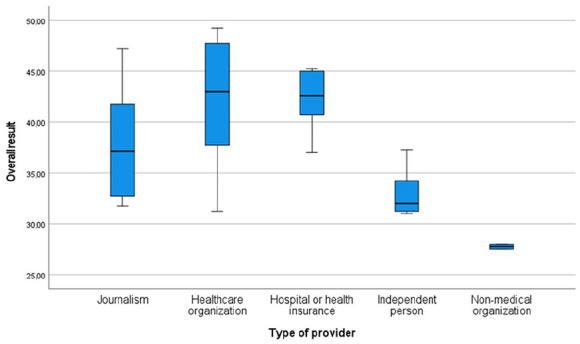
Overall results of the videos in points depending on the different types of providers.

Looking at the overall results of the individual videos, the lowest possible score was 20 points, the highest possible score was 60 points. The overall results of the videos are shown in brackets as percentages. Zero percent corresponds to the minimum achievable result of 20 points, 100% corresponds to the maximum achievable result of 60 points. The videos achieved a mean overall result of 38.2 points (45.4%) and a standard deviation of 6.5 points, a minimum of 27.5 points (18.8%) and a maximum of 49.3 points (73.1%). The standard deviation in points was 6.5. Classifying the videos in 4 levels from very poor (0% -25%), poor (25%-50%), good (50%-75%) to very good (75%-100%), no video achieved a rating of very good. Twelve videos were good, eleven poor and 2 very poor. More detailed information can be found in Supplemental Table 6. None of the videos met all the criteria. The overall result correlated positively with the video length (Spearmans ρ = 0.43, *P* = .03), but not with the number of likes, comments, and views. The highest mean was found in the group “ hospital or health insurance” with 42.2 points (55.5%), the second highest in “healthcare organization” with 42.0 points (55%), followed by “journalism” with 38.0 points (44.9%), then “independent person” with 33.2 points (32.9%) and “non-medical organization” with 27.8 points (19.4%) (Figure 2). Nevertheless, Kruskal Wallis test showed no significant correlation between the overall result and the type of provider.

### The Role of the Search Term

If the search term is taken into consideration, 4 of the 5 videos with the highest overall result came from the search “CM” and 3 of the 5 videos with the lowest overall result came from the search “AM.” If we take a closer look at the overall result of the individual search terms, we see that “AM” had a mean of 35.1 points (37.7%) and a standard deviation of 6.5 points. “CM” had a mean of 40.6 points (51.5%) and a standard deviation of 5.6 points. Videos displayed with the search term “CM” had a significantly better overall result than videos displayed with the search term “AM” (Mann-Whitney U test: *U* = 39.5, *P* = .04). However, these videos were watched less frequently (*U* = 31.0, *P* = .01) and had fewer likes (*U* = 35.0, *P* = .02) and comments (*U* = 27.0, *P* = .01) per views. No significant association was found regarding the video length.

### Individual Criteria

Looking at the individual criteria (Table 4), the lowest mean value of all rated videos was recorded for the criteria “presentation of numbers and outcomes” and “quality assurance procedure.” The highest mean value was recorded for the criterion “audio quality.” Videos with a higher scoring for “evidence-based information” had a higher score in “communication of missing evidence” (Spearman’s ρ = 0.85, *P* < .001). This criterion asks whether missing evidence is openly communicated in the video.

**Table 4.** Overview of the Average Results of All Videos in the Individual Criteria. (The Lowest Possible Score Per Criterion Was One, the Highest Was Three).

	Mean [points]	Standard deviation [points]
Goal	2.1	0.6
Clarity	1.8	0.6
Comprehensibility of structure	2.0	0.6
Comprehensibility of language	2.6	0.3
Detailed information about treatment methods	1.7	0.7
Completeness	1.8	0.6
Accuracy	1.5	0.5
Observance of scientific knowledge on the presentation of numbers and outcomes	1.1	0.2
Evidence-based information	2.0	0.7
Communication of missing evidence	1.7	0.7
Relevance	2.8	0.3
Feasibility	2.0	0.6
Interaction with users	1.9	0.4
Video description	2.2	0.5
Transparency	1.7	0.4
References	1.4	0.5
Quality assurance procedure	1.1	0.1
Pointing out the limitations of the medium	1.5	0.6
Audio quality	2.8	0.2
Visual quality	2.7	0.5

Furthermore, videos with a higher scoring in the overall result of the individual videos had a higher score for the criteria “evidence-based information” (Spearman’s ρ = 0.86, *P* < .001) and “communication of missing evidence” (Spearman’s ρ = 0.89, *P* < .001).

## Discussion

YouTube being the most used video platform worldwide, enables the provision of educational and helpful information. Nevertheless, studies have reported that health-related videos are mostly of poor quality and even contain misleading information.^45-48^ Former studies assessed the quality of health-related videos, referring to surgeries^49^ or different types of cancer, such as prostate cancer^50,51^ and pancreatic cancer.^52^ To the best of our knowledge, this study is the first to address the topic of CAM in cancer care on YouTube.

Summarizing our results, we created an assessment tool that can be applied to all health-related video content and showed a good to excellent interrater reliability. The average video result was of poor quality. Videos found by searching for the term “CM” were of better quality than videos found by searching for the term “AM.” An orientation toward the interaction indices did not help to identify high-quality videos. Videos from journalistic sources were the most watched, but there was no correlation between the provider type and the overall result of the individual videos.

### Usability of the Assessment Tool

To our knowledge and after thorough search of the literature, no standardized assessment tool for video assessment that is not focused on a single, specific health topic existed at the time our assessment tool was created, and the videos were selected. In most cases, the authors combined existing tools from the field of evaluation of written information. The most commonly used tools are: PEMAT, DISCERN, GQS (Global Quality Scale), VPI (Video Power Index) and Misinformation Scale.^45,48,50,52^ However, several surveys have already critically questioned the transferability of these tools to YouTube videos and called for the development of a standardized protocol.^53-55^ They criticize that DISCERN and JAMA score were both designed for web pages and thus cannot be applied to videos. Moreover, they were both designed before the development of YouTube.

As we did not find an adequate tool for our purpose, we decided to create a new instrument from the already existing tools based on our working group’s experience with the analysis of other types of information material. The instrument was also used in a parallel study, in which Grumt et al^56^ assessed YouTube videos on nutrition in cancer care (publication submitted).

According to the ICC, the tool showed good to excellent interrater reliability. The ICC in general presented itself as particularly good compared to other studies with comparable to significantly worse results (ICC = 0.69 - 0.9).^57-59^ As we didn’t find a relevant difference between the ICC of the 2 rater groups with a different knowledge level, the assessment tool is made for experts as well as students to evaluate health-related videos. The question remains whether the tool is also suitable for laypeople. On the one hand, they may lack the knowledge to be able to assess the level of evidence of the information in the video. On the other hand, it is not guaranteed that we as professionals can adequately empathize with the perception and comprehension skills of laypeople. This could have an influence on the overall result of the videos, particularly in the case of the 2 criteria “comprehensibility of language” and “relevance.” As this aspect only relates to a few criteria, no major bias in the results is to be expected.

To identify a video as high quality, we believe that ensuring evidence is the most important criterion to prevent the spread of misinformation and providing good clinical guidance. In this context, the high correlations between the overall result of the individual videos and the 2 sub-criteria relating to evidence confirmed the functionality of our assessment tool.

It is important to discuss that an article with a similar tool was published after the development of our assessment tool. Guler et Aydin^55^ set out to design a measurement tool that would allow both laypeople and experts to rate medical videos of all kinds. For this purpose, they developed a tool with 15 items. 10 videos with different medical topics were rated by 25 medical and 25 non-medical participants using a 5-point Likert scale. Even though the results are significant and definitely help us to evaluate the data situation on YouTube, we still see some gaps in the tool provided and would have revised it further for our analysis if it had already been available during our research work. The most important aspect for us, which is missing in the tool from Guler et Aydin, is the control of the evidence. In our opinion, this is immensely important for evaluating a video. Of course, this makes it more difficult for laypeople to evaluate, but this is not addressed in the article. Evidence may be particularly important in the field of CAM, as there is a lot of misinformation circulating, but evidence must also be checked for all other medical topics. In addition, a comprehensive consideration of the topic, including the risks and effects of certain treatment options, is also important for a high-quality video. In the developed tool, no query of completeness and critical consideration is guaranteed for the items. In addition, the tool does not clarify whether limitations are communicated in the video and, for example, whether consultation with the supervising doctor is recommended. In comparison with our tool, further differences are noticeable, making our tool more comprehensive, but possibly also more demanding to use.

### Quality of the Videos

The quality of the videos in our study was mainly between good and poor and no video met all the criteria. The average video result was of poor quality. García-Cano-Fernández et al,^47^ Yurdaisik,^45^ Steinberg et al^46^ and Di Bello et al^48^ also described the average quality of videos on bladder, breast, prostate and testicular cancer as poor.

Videos found by searching for the term “CM” led to significantly better results than videos found by searching for the term “AM.” It makes sense that content that uses a more technical term (CM) is more likely to be created by professionals and therefore tends to be of higher quality. Furthermore, all videos that were from the type of provider “hospital or health insurance” which showed the highest mean overall result came from the search “CM.” This confirms that professionals tend to use more technical language (CM). On the other side, our study showed that videos found by the search “CM” were viewed less frequently and had less likes and comments than videos found by the search “AM.” One reason for this could be that users more often use “AM” as a search term, because they do not know the more technical term.^60^ Another reason for this could be that patients are primarily searching for alternatives to their treatment and therefore come across the videos that are displayed with the search term “AM”. In this case, research work like ours would be even more important to ensure that patients are not misguided in their search for information.

Furthermore, videos from the provider type “journalism” were viewed the most and significantly more frequently than videos from the provider group “hospital and health insurance.” A reason for this could be that journalistic providers have a particularly large reach due to a wider content field they refer to and a larger number of followers. This shows that not only higher quality videos are needed, but also that the reach of these videos must be improved. We recommend increased collaboration between healthcare institutions and journalistic media to increase the reach of high-quality videos. Users are advised to search for videos using technical terms to get the best possible results. Since laypeople might have difficulties with technical terms, it could help to tag videos that are currently found by the search “CM” with the keyword “AM” so that they are also displayed under this search term.

### Recognizing High-Quality Videos

In contrast to user expectation, interaction indices (likes, comments, views, view ratio) were not adequate to identify high-quality videos. Steinberg et al^46^ who examined videos on prostate cancer showed the same result. Therefore, orientation on the interaction indices is not recommended.

In line with Sahin et al,^57^ Cassidy et al^58^ and Steinberg et al^46^ we found that videos with a higher overall score had significantly longer runtimes than videos with a lower score. This finding is reasonable, as longer videos offer more time to explain facts in more detail. The concern that longer videos might be watched less frequently wasn’t confirmed in our study. Shungu et al^59^ even found an association between longer videos and a higher number of views per month in their study on prostate cancer screening for black men. Consequently, we recommend producing longer videos if this makes them more detailed.

The videos of the 2 types of providers from the healthcare segment had the highest overall result on average. However, there was no significant difference in the rating between the types of providers. Grumt et al^56^ using the same assessment tool, found that videos from hospitals and healthcare organizations (registered non-profit organizations) were rated significantly better than videos from independent persons (laypeople and individual professionals not operating in the context of an official organization). The lack of significance in our study is likely due to the small number of videos per type of provider.

The difficulty of identifying credible sources in the health sector on social media was also realized by the WHO^61^ and YouTube.^62^ Therefore, shortly after our evaluation, YouTube initiated suggesting videos from the healthcare sector with existing standardized review mechanisms. To identify those videos, YouTube follows principles and definitions drafted and reviewed by the NAM (National Academy of Medicine) and APHA (American Public Health Association) and follows the work of the WHO and CMSS (Council od Medical Speciality Societies). As a result, the videos suggested for the term “CM” include 3 that are among the 8 best videos according to our ratings. Focusing on reliable sources is an effective first step. Nevertheless, the strategy could be improved, since videos from providers that do not fulfill the specific criteria or aren’t checked, but present themselves as high-quality and reliable, are not labeled yet. One example is the video from the self-help group “Hautkrebs Netzwerk Deutschland e.V., which was the best video in our evaluation, but wasn’t labeled by YouTube. Therefore, YouTube provides further authorization tests, that aren’t available in all countries yet, but show great future potential. The critical question will be whether and how YouTube will organize the further implementation and guarantee reliable classification in view of the gigantic number of providers and videos across the world. This becomes even more important in view of studies that have shown a still rather low knowledge and use of CAM among medically underserved cancer patients with a simultaneous high level of interest but low level of education.^63^

## Conclusion

Overall, the quality of the YouTube videos was roughly balanced between good and poor, but no video reached the top quarter of rating. Since videos of the provider type journalism were by far the most viewed, a better cooperation between healthcare and media institutions is recommended to enable a new quality of health-related patient education. Here, the labeling of videos from reliable sources in the health sector introduced by YouTube is a promising development which should be scientifically monitored in the future.

The developed assessment tool is more detailed than any previously developed method for video rating in the medical field. Further testing and development of the method toward a standard for the evaluation of health-related videos is recommended. Particularly when it comes to decisions that affect human health, a good, scientifically proven basis of information is essential.

## Limitations

The study has several limitations. We only included German-language videos and limited the number of videos to a maximum of 30, which does not correspond to the full scope of the data. Although the selection of rated videos roughly represents the distribution, the small number makes it difficult to draw conclusions on differences in quality among the types of providers. Since YouTube is a rapidly developing platform, its data is also constantly evolving, so that a statement about the quality of the videos provided is a snapshot. Time has also passed since our assessment tool was created and the article was written, and another assessment tool has been published in the meantime. If the research work had taken place at a later date, there might have been an opportunity to review and expand the already existing tool. Nevertheless, there is now an opportunity to compare the 2 tools in future work and combine the best of both. People might criticize that only YouTube was used as a research platform, but as this is the most used, it is a good first step. Furthermore, our assessment tool has not yet been tested and may have set priorities by including or not including individual sub-criteria in the evaluation. However, due to the good to excellent ICC in our evaluation run, we can assume that the results are reliable.

## Supplemental Material

sj-docx-1-ict-10.1177_15347354241293417 – Supplemental material for Quality Assessment of YouTube Videos on Complementary and Alternative Medicine (CAM) for Cancer Using a Newly Developed Tool Supplemental material, sj-docx-1-ict-10.1177_15347354241293417 for Quality Assessment of YouTube Videos on Complementary and Alternative Medicine (CAM) for Cancer Using a Newly Developed Tool by Sophia Huchel, Alina Grumt, Christian Keinki, Judith Buentzel, Lukas K�smann and Jutta Huebner in Integrative Cancer Therapies

## Appendix

**Table A1.** Final Assessment Tool.

**Assessment tool for video rating** 1 point = The video barely meets the criterion. 2 points = The video misses the criterion in some points. 3 points = The video fulfills the criterion at its best.	Points
**Content criteria**	
**Goal:** Are the goals of the video clear? Does the video achieve its self-imposed goals?	① ② ③
**Clarity:** Is there an outline? Is the video broken down into sections that make sense in terms of content? Are headings used for sections? Is there a summary within the video? Are there visual elements that show where you are in the video?	① ② ③
**Comprehensibility of structure:** Does the order in which information is presented facilitate comprehension? Are pictures and graphics (e.g. diagrams, illustrations, etc.) used in a way that supports the spoken word?	① ② ③
**Comprehensibility of language:** Are the sentences short and clearly formulated? Are medical terms used only when it helps to understand or prepare for the medical consultation?	① ② ③
**Detailed information about treatment methods:** Does the publication describe the benefits (including quality of life), mode of action, and risks of each method?	① ② ③
**Completeness:** Is the topic completely covered? Is there information about supplementary help/info?	① ② ③
**Accuracy:** Are the statements clear and substantiated with the help of figures?	① ② ③
**Presentation of numbers and outcomes:** Does the presentation of numbers prevent the occurrence of misunderstandings? (Example: Better comprehensibility by using absolute numbers such as 10 out of 100 instead of 10%) or are pictorial representations used (to clarify numerical data)?	① ② ③
**Evidence-based information:** Is the information evidence-based?	① ② ③
**Communication of missing evidence:** Is missing evidence openly communicated?	① ② ③
**User-orientated criteria**	
**Relevance:** Is the information relevant to those affected?	① ② ③
**Feasibility:** Does the video clearly identify at least one action that users can perform? Does the video speak directly to users when describing the activity? Does the video break down each activity into manageable steps? Does it use language that helps users understand the topic and enables them to talk to the clinician about it (participation-supporting language)?	① ② ③
**Interaction with users:** Is there a possibility for feedback and questions from users within and/or outside the comment function? (E.g. is a link available, was there a reaction to comments?)	① ② ③
**Video description:** Does the video title match the video content? Does the video description provide a summary of the video content? Is the focus of the video identifiable?	① ② ③
**Formal criteria**	
**Transparency:** Is the following information disclosed: Authorship, provider information, funding (e.g. sponsors), advertising policy (Is advertising clearly distinguished from editorial content?)?	① ② ③
**References:** Is it communicated in the video or in the video description from which scientific sources the information in the video originates? Is it communicated in the video or in the video description when this presented information was created?	① ② ③
**Quality assurance procedure:** Is it clear whether the validity is checked and an update is made if necessary? Is it clear how the quality of the video was checked? (Seal, experts of the professional society)	① ② ③
**Pointing out limitations of the medium:** Is it pointed out that the video is not a substitute for a doctor’s visit?	① ② ③
**Audio quality:** Is background noise avoided? Is there supporting background music, signal tones or similar? Is the volume window appropriate? Is the speed of speech appropriate? Is the speaker easy to understand?	① ② ③
**Visual quality:** Are objects and people well illuminated? Is the image sharp? Is the camera movement appropriate? Is the text in the video easy to read?	① ② ③
**Total score**	
