# Supplementary material for: Quality Assessment of YouTube Videos on Complementary and Alternative Medicine (CAM) for Cancer Using a Newly Developed Tool
Source: Integr Cancer Ther. 2024 Oct 28;23:15347354241293417. doi: 10.1177/15347354241293417 (PMC11528743; doi:10.1177/15347354241293417)
Supplement: sj-docx-1-ict-10.1177_15347354241293417 – Supplemental material for Quality Assessment of YouTube Videos on Complementary and Alternative Medicine (CAM) for Cancer Using a Newly Developed Tool [file sj-docx-1-ict-10.1177_15347354241293417.docx]

Supplementary material

**Table 6.** All rated videos with their original title, type of provider, provider name, overall result and results within the three main criteria (content, user-orientated and formal criteria) in percent and in points.

| **Video title** | **Type of provider** | **Overall result in % [in points]** | **Content criteria in % [in points]** | **User-orientated criteria in % [in points]** | **Formal criteria in % [in points]** |
| --- | --- | --- | --- | --- | --- |
| Komplementäre Medizin in der Onkologie | Healthcare organization  (Hautkrebs-Netzwerk Deutschland e. V.) | 73.1  [49.3] | 77.5 [25.5] | 84.4 [10.8] | 58.3 [13.0] |
| Komplementäre Medizin bei Krebs – wissenschaftliche Erkenntnisse und praktische Anwendungen | Healthcare organization (Krebsinformationsdienst des Deutschen Krebsforschungszentrums) | 69.4 [47.8] | 70.0 [24.0] | 81.3 [10.5] | 60.4 [13.3] |
| Krebs und Komplementärmedizin: Patientenwebinar am Samstag im Rahmen der Feldkircher Onkologietage | Journalism  (Vorarlberger Nachrichten) | 68.1 [47.3] | 75.0 [25.0] | 65.6 [9.3] | 58.3 [13.0] |
| Patiententag "Leben mit Krebs" - Komplementärmedizin: Was ich selbst gegen Krebs tun kann | Hospital or health insurance  (BBTGruppe Region Tauberfranken-Hohenlohe) | 63.1 [45.3] | 67.5 [23.5] | 78.1 [10.3] | 45.8 [11.5] |
| Komplementärmedizin für Krebspatienten - Teil 4: Krebs und Immunsystem - Immunstimulanzien, Mistel | Hospital or health insurance  (Die Techniker) | 62.5 [45.0] | 71.3 [24.3] | 62.5 [9.0] | 47.9 [11.8] |
| CALL-IN \| (K)ein Kraut gegen den Krebs? Wie Komplementärmedizin Tumorpatienten helfen kann | Healthcare organization  (yeswecan!cer) | 62.5 [45.0] | 58.8 [21.8] | 84.4 [10.8] | 54.2 [12.5] |
| FORUM «Wie kann die Komplementärmedizin bei Krebs unterstützen » Dr Matthias Rostock | Hospital or health insurance  (Universitätsspital Zürich) | 56.9 [42.8] | 53.8 [20.8] | 62.5 [9.0] | 58.3 [13.0] |
| Komplementäre Behandlungsmethoden bei Krebserkrankungen | Hospital or health insurance  (Universitätsklinikum Freiburg) | 56.3 [42.5] | 55.0 [21.0] | 78.1 [10.3] | 43.8 [11.3] |
| Homöopathischer Hokuspokus: wenn der Glaube an Alternativmedizin tödlich endet \| SPIEGEL TV | Journalism  (DER SPIEGEL) | 54.4 [41.8] | 48.8 [19.8] | 71.9 [9.8] | 52.1 [12.3] |
| Komplementärmedizin beim Immunsystem | Healthcare organization  (CancerSurvivor – Menschen mit Krebs) | 52.5 [41.0] | 51.3 [20.3] | 68.8 [9.5] | 43.8 [11.3] |
| FORUM «Wie kann die Komplementärmedizin bei Krebs unterstützen » Prof Dr Claudia Witt | Hospital or health insurance  (Universitätsspital Zürich) | 51.9 [40.8] | 53.8 [20.8] | 59.4 [8.8] | 43.8 [11.3] |
| Aprikosenkerne gegen Krebs: Lebensgefährliche Naturheilkunde \| SPIEGEL TV | Journalism  (DER SPIEGEL) | 50.6 [40.3] | 47.5 [19.5] | 71.9 [9.8] | 41.7 [11.0] |
| Brustkrebsinfotag 2021: Komplementärmedizin Ergänzende Unterstützung f. Frauen mit & nach Brustkrebs | Healthcare organization  (Brustkrebs Deutschland e.V.) | 44.4 [37.8] | 30.0 [16.0] | 71.9 [9.8] | 50.0 [12.0] |
| Naturheilkunde bei Krebs – Wie sinnvoll ist das? | Independent person  (naturheilzentrum bottrop) | 43.1 [37.3] | 30.0 [16.0] | 71.9 [9.8] | 45.8 [11.5] |
| FORUM «Wie kann die Komplementärmedizin bei Krebs unterstützen » Lena Kümmel | Hospital or health insurance  (Universitätsspital Zürich) | 42.5 [37.0] | 42.5 [18.5] | 50.0 [8.0] | 37.5 [10.5] |
| Komplementärmedizin in der Onkologie Teil 1 | Independent person  (Bernhard Dr. med. Ost) | 35.6 [34.3] | 27.5 [15.5] | 34.4 [6.8] | 50.0 [12.0] |
| Methadon gegen Krebs? - Die ganze Reportage \| stern TV (21.06.2017) | Journalism  (stern TV) | 35.0 [34.0] | 22.5 [14.5] | 53.1 [8.3] | 43.8 [11.3] |
| Die 3 Hauptursachen von Krebs \| Krebsforscher Lothar Hirneise \| NaturMEDIZIN \| QS24 | Journalism  (QS24 - Schweizer Gesundheitsfernsehen) | 31.9 [32.8] | 20.0 [14.0] | 59.4 [8.8] | 33.3 [10.0] |
| »Krebs ist schon lange heilbar.« \| Chemo-Gegner*innen, auf ein Wort. | Independent person (Zimtkopfliest) | 30.0 [32.0] | 22.5 [14.5] | 46.9 [7.8] | 31.3 [9.8] |
| Die versteckten Ursachen hinter Krebs \| Dr. med. Thomas Rau \| NaturMEDIZIN \| QS24 | Journalism  (QS24 - Schweizer Gesundheitsfernsehen) | 29.4 [31.8] | 15.0 [13.0] | 56.3 [8.5] | 35.4 [10.3] |
| KrebsLeben und mentaler Einfluss auf Heilung - Spitzen-Gespräch Miriam Reichel & Prof. Jörg Spitz | Healthcare organization  (Akademie für menschliche Medizin) | 28.1 [31.3] | 13.8 [12.8] | 56.3 [8.5] | 33.3 [10.0] |
| 004 Komplementärmedizin bei Krebs: Immunsystem. Schlafplatzhygiene. | Independent person  (Jürgen Dahn) | 28.1 [31.3] | 18.8 [13.8] | 46.9 [7.8] | 31.3 [9.8] |
| Dr. Petra Wiechel Komplementärmedizin Tumore Krebs Paracelsus Clinica al Ronc | Independent person  (Ernst Crameri) | 27.5 [31.0] | 13.8 [12.8] | 53.1 [8.3] | 33.3 [10.0] |
| PROGNOS- Kongress: Krebs & Alternativmedizin von Dr. med. Johannes Wilkens | Non-medical organization  (Recht auf Durchblick) | 20.0 [28.0] | 15.0 [13.0] | 34.4 [6.8] | 18.8 [8.3] |
| Krebs und Lebensqualität unter ganzheitlicher naturheilkundlicher Therapie – Alternativmedizin | Non-medical organization  (VBN Verlag Lübeck) | 18.8 [27.5] | 11.3 [12.3] | 40.6 [7.3] | 16.7 [8.0] |
